# Book Review

**Published:** 1903-12

**Authors:** 


**Sir Henry Wentworth Acland, Bart., K.C.B., F.R.S.** A Memoir. By J. B. Atley. Pp. viii., 507. London: Smith, Elder & Co. 1903.

All Oxford medical graduates, as well as many Oxford men unconnected with medicine or even science, will read with interest Mr. Atley's biography of the late Regius Professor of Medicine. Sir Henry had been for so many years a prominent man in the University, had taken a leading part in placing the teaching of science on its present sure foundation, and was well known, we might almost say, as the father of the Museum. The many interesting facts brought together in the volume before us reflect great credit on the author, who has shown that he can be a painstaking biographer, and write a book which does not contain a dull place between its covers.

Sir Henry Acland was born to achieve greatness, and would have made his mark in whatever profession he had thrown his lot. The son of a well-known west-country baronet, the relative of distinguished persons, gifted with a handsome and commanding presence, possessed of charming manner, gifted with no mean powers of oratory, and with a kind and sympathetic nature, it would have been surprising if he had not risen to the position he did even in a profession that did not generally command respect or obtain social recognition in the forties of the last century. Sir Henry's reputation, however, did not rest on his success as a physician, though he may have done good work in the small sphere of a provincial city of about 30,000 inhabitants. It was gained by his advocacy of science as a part of the curriculum for the Arts degree at Oxford, and the great efforts he made, resulting in complete success, for the erection of the centre of scientific teaching—the Museum.

Sir Henry's connection with Oxford began in 1834, when he matriculated at Christ Church. Having spent the usual term of years here he, much to the surprise and chagrin of his relatives, selected medicine as his profession, and entered at St. George's, then still under the ægis of the late John Hunter. A short stay was made at Edinburgh before Oxford saw him again as Lee Reader in Anatomy, an old benefaction with a small salary and smaller duties. Up to this time Sir Henry had taken no qualification, but a favourable opportunity occurring to commence practice by the retirement of Dr. Wootten, he took the M.B. and diploma of the College of Physicians in 1846, both of which examinations were more or less of a farce, and moved to Broad Street, where a long line of Oxford men will remember him. In 1857, by the death of Dr. Ogle, the Regius Professorship became vacant, and on Lord Palmerston offering it to Sir Henry he at once accepted it, being subsequently appointed to the Clinical Professorship after a spirited fight. These Professorships he held for 37 years. Sir Henry's contributions to literature have chiefly a hygienic tendency, and we do not recall that any use was made of the Clinical Professorship to teach clinically, and it was no doubt chiefly due to this that the attack was made on Sir Henry's methods by the late editor of the *British Medical Journal.* The Regius Professor, however, had his own views of his duties and the possibilities of a Teaching Medical School at Oxford, and was able not only to carry many leading men with him, but also to convince the Commissioners. The fact, however, remains that Oxford does not attract so many men who intend to take medical degrees as her sister University, though she is now making rapid strides to cover the distance lost. Of Sir Henry's tenure of the Chairmanship of the General Medical Council little need be said. He attended regularly, missing only one meeting in nine years, and conducted the business with tact and firmness. We need not here refer to the lost opportunities of the Council or its inability to represent the great body of practitioners in the kingdom; they have little to do with Sir Henry or Mr. Atley.

Coming then to less debatable matters, we can give our whole-hearted praise for Sir Henry's efforts on behalf of the Museum and scientific training as conducted in the University. He was the leading spirit in the movement, and there is not the smallest doubt that the collection of the money and the subsequent erection, on the somewhat fantastic design of Woodward, of the Museum was largely due to him. This will be a far more lasting memorial of Sir Henry Acland than his strictly professional work, and too much praise cannot be accorded to him for the strenuous efforts on behalf of the scheme. He lived to see his project carried out, and no doubt far beyond his expectations.

Mr. Atley has done his part well. He has written a very interesting book, and treated many difficult matters with delicacy and fairness. Not an unkind word can be found even on subjects about which many were said and written. The index is merely one for names, which is a pity, and the dates on the headings of the pages are often confusing, but these are small matters.

**Medico-Chirurgical Transactions.** Vol. LXXXVI. London: Longmans, Green & Co. 1903.—A noteworthy paper by Dr. Cecil Wall on acute cerebro-spinal meningitis, caused by the diplococcus intracellularis of Weichselbaum, forms a complete clinical study of this disease, which appears to have been fairly frequent of late, as shown by the fact that a series of thirty-eight cases forms the foundation of the study. The utility of the Kernig sign is indicated, but little is said of the absence of the Widal reaction as giving an early means of differentiation from enteric fever. Professor T. Clifford Allbutt, F.R.S., contributes a most useful paper on the rise of blood pressure in later life.

**The Medical Examination for Life Assurance.** By F. DE HAVILLAND HALL, M.D. Third Edition. Pp. 112. Bristol: John Wright & Co. 1903.—Such a compact guide for medical examinations in Life Assurance meets the difficulties that always present themselves to the novice in this special and technical aspect of medical practice. It is the work of an expert, is reliable, and above all handy. The actuarial tables and the rules observed by various life assurance societies are not in any sense empirical, but are based on accumulated experiences, and it is instructive to observe how widely different current impressions are from the actual facts that such experience reveals on many questions. For instance, "an attack of acute rheumatism sufficiently severe to keep the patient in bed two or three weeks, and to incapacitate him from his occupation for six or seven weeks, would require the addition of an equivalent of seven years at the age of thirty, and three years must have elapsed since the attack. If there has been more than one attack, the life would not be assurable until at least ten years after the last attack. An applicant with a rheumatic history had better not be accepted under the age of twenty-five years." These rules are shown by the report on "The causes of death among the assured in the Scottish Widows' Fund" to be by no means too stringent, but probably few medical practitioners would have come to such conclusions from personal experience alone. The work is full of the most useful suggestions and information.

**Transactions of the Medical Society of the State of New York for the Year 1903.** Albany: Brandow Printing Company.—The principal feature of this volume is a series of papers entitled "A Symposium on Arterio-sclerosis." The relation of arterio-sclerosis to the heart is described by Dr. Butler, to the kidney by Dr. Lyon, to the digestive system by Dr. Stockton, to the nervous system by Dr. Browning, whilst the pulsus infrequens is described by Dr. Satterthwaite, and the early diagnosis and general symptoms of arterio-sclerosis are summarised by Dr. Rochester.

**Medico-Psychological Association of Great Britain and Ireland. "A Revision of the Statistics presented by the Committee on Tuberculosis."** By T. A. Chapman, M.D. London: Adlard and Son. 1903.—Dr. Chapman has revised the figures in the report of 1902, but does not in any way traverse the conclusions then arrived at, viz.: that tuberculosis in asylums is largely due to infection, to damp and ill-drained sites, and to inadequacy of time spent out of doors, of cubic space indoors, and of means of artificial ventilation.

**St. Bartholomew's Hospital Reports.** Vol. XXXVIII. London: Smith, Elder & Co. 1903.—This volume is somewhat disappointing. It is well bound and of imposing size, and one expects much from its appearance, and the fact that it represents, or should do so, the amassed experience of a year's work in one of the largest London hospitals, but half the volume is devoted to the catalogue of specimens added during the year to the museum, and to the synopsis of the annual case books. There are but few articles from the pen of the staff, and these confined to a few interesting cases. This year there is an excellent article on glioma of the retina by W. H. Jessop, which also appeared in the Transactions of the British Medical Association in 1901. A report on some experiments concerning the causation of the first sound of the heart by O. Inchley shows evidence of a painstaking original research, and goes to prove that the sound in question is due to the admixture of two sounds, the first due to and synchronous with auricular systole, and the second beginning with ventricular systole when the valves are already closed, the initial sound being altered in mitral stenosis and the other in regurgitation. The rest of the articles are for the most part the narration of interesting or uncommon cases.

**King's College Hospital Reports.** Vol. VIII. 1901.—The history of the College and Hospital has come to a premature termination by the decease of the writer, Professor John Curnow, whose obituary notice is the most noteworthy feature of the volume. "There never was a man who talked and looked less like the popular conception of the resourceful physician or studious man of science. . . . It is superfluous to say that his abilities were admired and he himself was liked by all who knew him, and his death leaves a gap in our ranks which is, from a social point of view, most serious." An article on nephritis in children by Dr. George F. Still calls attention to the fact that "there is no age at which nephritis may not occur." The author does not, however, direct attention to the fact that hæmaturia in infants may be due to scorbutic conditions, dependent on dietetic defects, and easily cured as soon as the cause is recognised.

**Saunders' Year-Book of Medicine and Surgery.** 1901, 1902 and 1903. London: W. B. Saunders & Company.—The editorial staff, under the general charge of Dr. George M. Gould, may be congratulated on the increasing popularity of this year-book, and in the experiment made first in 1900 of issuing the work in two volumes, one of medicine and one of surgery. This experiment has proved to be very acceptable to subscribers, and it has therefore been continued. Each volume contains about 700 pages of well-printed matter, and is a carefully prepared yearly digest of scientific progress. The names on the editorial staff are a guarantee that the work will be well done, and on perusal of the volume it is difficult to discover any inaccuracies or serious omissions.

**Index-Catalogue of the Library of the Surgeon-General's Office, United States Army.** Second series. Vol. VIII.—*Insane—Kysthospitalet.* Pp. 894. Washington: Government Printing Office. 1903.—The large amount of labour attended with the production of this indispensable work is shown by the fact that the present volume contains 10,704 author-titles, 5,731 subject-titles of separate books and pamphlets, and 29,684 titles of articles in periodicals. The War Department of the United States may well be congratulated on the thorough manner in which these volumes are prepared, and without which no medical library can be considered complete.

**Reports from the Laboratory of the Royal College of Physicians, Edinburgh.** Vol. VIII. Edinburgh: Oliver & Boyd. 1903.—This volume contains so many excellent papers and contributions to various branches of physiology and pathology, that no summary even can be attempted. Nearly a third of the volume is taken up by the record of an elaborate research, of the greatest economic and social importance, of the energy-value and prices of diet of the labouring classes in Edinburgh—investigation of which will undoubtedly play a great part in the discussion of the economic condition of the working classes in this country. The other notable paper is by Chipman on the development of the placenta of the rabbit, in which many disputed questions are apparently set at rest.

**Diseases of the Skin.** By MALCOLM MORRIS. New [Third] Edition. Pp. xvi., 642. London: Cassell & Co., Ltd. 1903.—

We have had the opportunity of reviewing this work—when the revised edition appeared in 1898—and also an edition in 1894. There is no doubt that works on Dermatology have increased in importance: there is an increasing demand for them, and therefore we cannot be surprised that the volume of this work has likewise become augmented, and that 614 pages of well and carefully-printed letterpress are required to do the duty of 578 pages in the previous edition, and that the Index should have developed into twenty-seven good-typed pages. Speaking generally, the present work seems well up-to-date, which we are sure Mr. Morris carefully aims at, and therefore it seems almost superfluous to enter into details. Sabouraud's researches on Ringworm are carefully stated, and the various views concerning the invading parasite, judiciously weighed. We should have wished for more enlightenment on the light treatment of lupus, and still more especially on the value of X-rays in rodent ulcer, and we have not noted a word about the help derived from high-frequency currents in the treatment of several skin affections. We notice that a marked preponderance (p. 160) of right-sided Herpes Zoster is still claimed. We have gone into this point, with large statistics, and we question this marked incidence.

**The Etiology of Typhoid Fever and its Prevention.** By W. H. Corfield, M.D. Pp. xv., 159. London: H. K. Lewis. 1902.—These Lectures, being the Milroy Lectures delivered at the Royal College of Physicians in 1902, give in concise form an admirable summary of the various factors concerned in the causation of typhoid fever. The information is well up-to-date, and the volume will well repay careful perusal.

**Papers and Reports of the American Public Health Association.** Vol. XXVII. Columbus, Ohio. 1902.—The sixty or seventy separate contributions to this volume present wide and varied reading in questions affecting the public health. We would call special attention to the article by Drs. Walter Reed and James Carroll on the prevention of yellow fever, with numerous illustrative plates of the mosquito (Stegomyia Fasciata) so prominently associated with the spread of this disease. The other contributions maintain a high level of interest.

**Public Health Laboratory Work.** By Henry R. Kenwood, M.B. Third Edition. Pp. xv., 606. London: H. K. Lewis, 1903.—The third edition of this well-known laboratory manual has been largely rewritten and thoroughly brought up-to-date. An appendix by Dr. W. G. Savage is added dealing with the bacteriological examination of water, milk, soil, etc. The book will prove of the greatest service to candidates preparing for Public Health Examinations.

**Practical Pharmacy and Prescribing.** By James Calvert, B.A., B.Sc., M.D. Second Edition. Pp. 110. London: H.

K. Lewis. 1903.—A most useful and comprehensive little work, which might with benefit be in the hands of every medical student, and would be almost equally useful on the shelves of the consulting room on entering practice. The question has often been debated whether the hospital pharmacopœia is a good or bad institution considered in the light of a factor in the education of the medical student in pharmacology and therapeutics. Probably the hospital formulary is indispensable as a saver of time for prescriber and pharmacist, but as its formulæ are necessarily generally compatible ones, the student when he begins his hospital work finds its extremely difficult to grasp the distinction between compatibles and incompatibles from a pharmaceutical standpoint. Dr. Calvert's little book furnishes a masterly and comprehensive key to the difficulties. Part I. gives a sketch of general dispensing methods and the various forms in which drugs are exhibited. The paragraph on page 2 on the weighing of small quantities of powerful drugs should quickly dispose of that apparently unsolvable problem—how to make twelve pills each containing say $\frac{1}{65}$ grain of strychnine with ordinary dispensing scales. The paragraphs on the constituents of drugs—tannin, starch, oils, etc.—form an indispensable preliminary to the study of incompatibles *per se*. One cannot well particularise where the whole of the examples are so universally useful, but as examples may perhaps be noticed, the paragraphs on sp. æther. nit. in conjunction with potass. iod. and the behaviour of quinine with various drugs, not forgetting the combination of quinine with sod. sal. which so repeatedly crops up. A very typical set of prescriptions exemplifying the various forms of incompatibility completes a particularly useful and practical book. It is often said that *practical* books on dispensing do not exist, a statement which is quite disproved by this work.

**Where shall I send my Patient?** A Guide for Medical Practitioners and Book of Reference to the Health Resorts and Institutions for Patients of Great Britain. Bournemouth: E. J. Frampton. 1903.—The association of medical men receiving resident patients has printed this volume, and are aware that this first number must have many omissions: their great desire is to make the book as complete and useful as possible, but they cannot do so without the co-operation of the authorities of institutions and hotels, who decline all invitations to send returns. The book contains lists of homes for all kinds of invalids, and a long list of houses where resident patients can be received. Particulars of these are to be obtained from the honorary secretary of the association, Bodorgan Manor, Bournemouth.

**Thirty-five Years at Contrexéville.** By Debout D'Estrées, M.D. Pp. viii., 132. London: The Health Resorts Bureau. 1903.—What should we do without uric acid? An excess of uric acid in the blood is the root of all evil, and the "cure" at Contrexéville eliminates it. There are other maladies besides uric acid, and other methods of cure, but of these we learn nothing from the author of this booklet of 132 pages. Certainly the author has improved on the peripatetic method of consultation in the park worthy of the more ancient Grecian philosophers.

**Buxton: its Waters, Baths, and Accessory Methods of Treatment.** By William Armstrong and J. E. Harburn. Pp. 71. Bristol: John Wright & Co. 1903. Fifty-six pages are a eulogy of the Buxton waters and baths as remedies of the greatest potency in the treatment of gout, rheumatism, and allied ailments. An appendix follows, consisting of short essays on Rheumatoid Arthritis, and the Nauheim treatment of Cardiac Diseases. The booklet appears to be a useful guide to those who are going to Buxton for treatment, and to medical men who are sending patients there.

**Atlas and Epitome of Fractures and Dislocations.** By Prof. Helferich. Edited by Dr. J. C. Bloodgood. Fifth Edition. **Atlas and Epitome of Abdominal Hernias.** By Dr. G. Sultan. Edited by Dr. Wm. B. Coley. Philadelphia and London: W. B. Saunders & Co. 1902.—We are glad to welcome these additions to the excellent series of hand atlases. Both volumes maintain the standard of production, and the occasional comments of the American editors in appropriate places greatly add to their value. The coloured illustrations are beautiful—often their beauty exceeds their real utility—and the general figures are numerous and good. The text is particularly characterised by the valuable inclusion of opinions and statistics from various sources, thus avoiding narrowness of appreciation. The atlas on fractures is brought well up-to-date. It necessarily omits many items of treatment familiar to English surgeons, and Sayre's method of treatment for fractured clavicle (p. 132) is inferior, we think, to the descriptions in English text-books. We hold a high opinion of the volume on Hernias, although the abdominal topography on p. 24, the employment of taxis with the patient standing erect (p. 108), and division of the stricture from without inwards (p. 119), is knowledge which might bring sorrow to a candidate at the Thames Embankment.

**Manuel de la Prostatectomie Périnéale pour Hypertrophie.** Par Robert Proust. Pp. 186. Paris: C. Naud. 1903.—This monograph is a model of good work, and characteristic of the literary care and artistic merit of our French colleagues in surgery. The surgical and pathological anatomy of the prostrate gland are expounded with a completeness we have never before met with, the indications for operation are described, and, lastly, a special perineal operation is given in full detail with beautiful illustrations. We can heartily recommend those interested in the subject to obtain this monograph and study it for themselves, for an adequate and yet condensed summary would be impossible.

